# Effect of lymphedema self-management group-based education compared with social network-based education on quality of life and fear of cancer recurrence in women with breast cancer: a randomized controlled clinical trial

**DOI:** 10.1007/s11136-020-02455-z

**Published:** 2020-03-09

**Authors:** Zahra Omidi, Masoomeh Kheirkhah, Jamileh Abolghasemi, Shahpar Haghighat

**Affiliations:** 1 Department of Reproductive Health and Midwifery, School of Nursing and Midwifery, Iran University of Medical Sciences, Tehran, Iran; 2 Nursing Care Research Center (NCRC), School of Nursing and Midwifery, Iran University of Medical Sciences, Tehran, Iran; 3 Department of Biostatistics, School of Public Health, Iran University of Medical Science, Tehran, Iran; 4 Breast Cancer Research Center, Motamed Cancer Institute, ACECR, Tehran, Iran

**Keywords:** Self-management, Lymphedema, Quality of life, Fear of cancer recurrence, Breast cancer

## Abstract

**Background:**

Breast cancer-related lymphedema (BCRL) and its associated symptoms harm the quality of life (QoL) of cancer survivors and can stimulate fear of cancer recurrence (FCR). Self-management education for lymphedema has been introduced as an effective method in controlling FCR. This study investigates the effect of lymphedema group-based education compared to the social network-based and control group on QoL and FCR in breast cancer patients.

**Methods:**

This three-arm clinical trial studied 105 patients with breast cancer-related lymphedema referred to Seyed_Khandan rehabilitation center. Sampling was done by random allocation method in blocks of 3 with 35 subjects in each group. All subjects received routine lymphedema treatments. The group-based education (GE) and social network-based education (SNE) groups received self-management education in the clinic and Telegram™ messenger channel, respectively. Impairment in QoL and mean score of FCR were assessed before, immediately after, and three months after the intervention by using the Persian version of Lymphedema Life Impact Scale (LLIS) and Fear of Progression Questionnaire-Short Form (FoPQ-SF), respectively. Mixed-model ANOVA was applied for statistical analysis.

**Results:**

There was a significant time effect on total LLIS (*P* = 0.007), psychosocial (*P* = 0.038) and functional (*P* = 0.024) subscale changes in three groups of study. Interaction between the main effect of group and time on psychosocial subscale changes was statistically significant (*P* = 0.017). The multicomparison results illustrated that the main effect of time, the main effect of group, and interaction of them on the mean score of FCR were *P* = 0.084, *P* = 0.380, and *P* = 0.568, respectively.

**Conclusion:**

Despite no significant reduction in the FCR score, results showed the improvement of most QoL aspects after three months of intervention. Although the social network-based education method was effective, the group-based education method was more beneficial. Applying these educational methods in lymphedema treatment protocols needs cost-effectiveness studies.

**Trial registration:**

This study was registered at the Iranian Registry of Clinical Trials (IRCT2017052834176N1).

## Introduction

Every year, 1.38 million breast cancer women are diagnosed in the world [1]. One of the most common side effects of breast cancer is lymphedema affecting about 40% of the survivors [2]. Lymphedema is the chronic and progressive accumulation of lymphatic fluid in the arm, breast, or trunk due to the impaired lymphatic circulation [3], and in addition to disfigurement, it is associated with disorders in psychosocial adaptation, functional status, quality of life (QoL), as well as economic problems [4, 5]. A systematic review showed reduced QoL as a result of decreased physical and psychological function and social well-being in women with lymphedema [6]. Swelling leads to physical discomfort like pain, heaviness, tightness, numbness, fatigue, and stiffness in the affected limb, which triggers the fear of cancer recurrence (FCR) [7].

FCR is defined as fear and concern about the return or progression of cancer in the same organ or any other parts of the body [8], affecting one-third of people with cancer [9]. Some degree of FCR is expected and adaptive (incentive to continue treatment). Although there is consensus that fear varies from normal to clinical level, there is no agreement on what constitutes the clinical level of fear [8]. High levels of fear lead to anxiety, dysfunction and reduce QoL of cancer survivors and even their caregivers. It can disrupt the everyday life of these people as a result of creating stress, difficulty in acceptance, psychological responses, functional disorders, physical disorders, as well as exacerbation of existing mental disorders [7, 8]. A high level of fear is a reason for avoiding referral to specialized cancer centers and continuing treatment [9] and produces disturbing thoughts and anxiety in women with lymphedema which evokes worry about their future [10]. FCR is one of the factors that reduce adherence to self-management behaviors in lymphedema [10–12]. Therefore, the expected results cannot be obtained from the treatment.

Complete decongestive therapy (CDT) consists of two phases of treatment. In the first phase, manual lymphatic drainage (MLD), compression garments, bandaging, exercise, and meticulous skincare are performed by a lymphedema specialist. Lymphedema self-management is the second phase (or maintenance phase) of standard lymphedema treatment. Self-management is necessary throughout life because lymphedema progresses without optimal management and the risk of ulcer and infection increases, negatively affecting an individual’s economy (due to increased costs of treatment), mental health and QoL [13–15]. However, the rate of adherence to lymphedema self-management behaviors is less than optimal, varying from 28 to 69% [13].

Self-management education can be used as a powerful tool in providing patients with information about caring skills along the cancer care chain [16, 17]. Self-management is often taught in a group and is associated with increased empowerment and self-confidence, facilitating positive relationships and social support [18]. The use of information and communication technology and the Internet, such as online social networks, provides cheaper and more cost-effective than in-person interventions due to lower costs of transportation, travel, equipment, and other overhead costs [19] and can reduce patient stress, improve treatment progress, empower individuals in self-management, increase individual’s participation in the treatment process, increase access to information, and improve QoL and self-efficacy skills [20–22]. Telegram, a messaging app, is one of the largest social networks with more than 200 million users worldwide, and with features such as channel allows broadcasting messages, photos, videos, and audio to an unlimited number of subscribers, and can be installed on a smartphone or laptop. Telegram can have functions in areas such as business or even patient education [23].

Despite the importance of lymphedema self-management, the focus of studies in the world and Iran is on self-management education of breast cancer patients during chemotherapy or immediately after treatment. While it seems benefits of self-management lymphedema, management of symptoms associated with lymphedema, besides to reducing of swelling [15], can be gained by using new tools such as Telegram, the most popular messenger in Iran [24]. Also, treatment outcomes typically assessed by objective measurement swelling or size, to indicate patients' perceived treatment benefit, need to subjective measurement [25]. Therefore, this study compares the effect of using different educational methods on QoL as one of the crucial aspects of lymphedema treatment and FCR as one of the most important concerns in survivor that there is no single approach to cope with it [26].

## Methods

### Trial design

This randomized, three-arm controlled clinical trial was conducted to compare the effects of group-based education and social network-based education on QoL and FCR in women with BCRL. The method of assigning individuals to groups was randomized blocks of three with the distribution of one individual in each group. Patients were randomly assigned to GE (*n* = 35), SNE (*n* = 35) and control (*n* = 35) groups. The Ethics Committee of Iran University of Medical Sciences approved the study (IR.IUMS.REC1395.9411173002).

### Participants

The inclusion criteria were a history of confirmed breast cancer (stages 0 to IV), lymphedema established by a physician in the past year, aged 18–65 years old, completion of primary cancer treatments, ability to read and write and work with the Telegram™ messenger, no post-cancer psychiatric disorders requiring drug therapy, and access to the Internet through cell phones or computers. The exclusion criteria were failure to attend in the third and fourth sessions of in-person education as the key sessions, failure to approve delivery of messages in the Telegram™ in the SNE group, detection of cancer recurrence during the study, and unwillingness to continue the intervention.

### Procedure

**Fig. 1:**
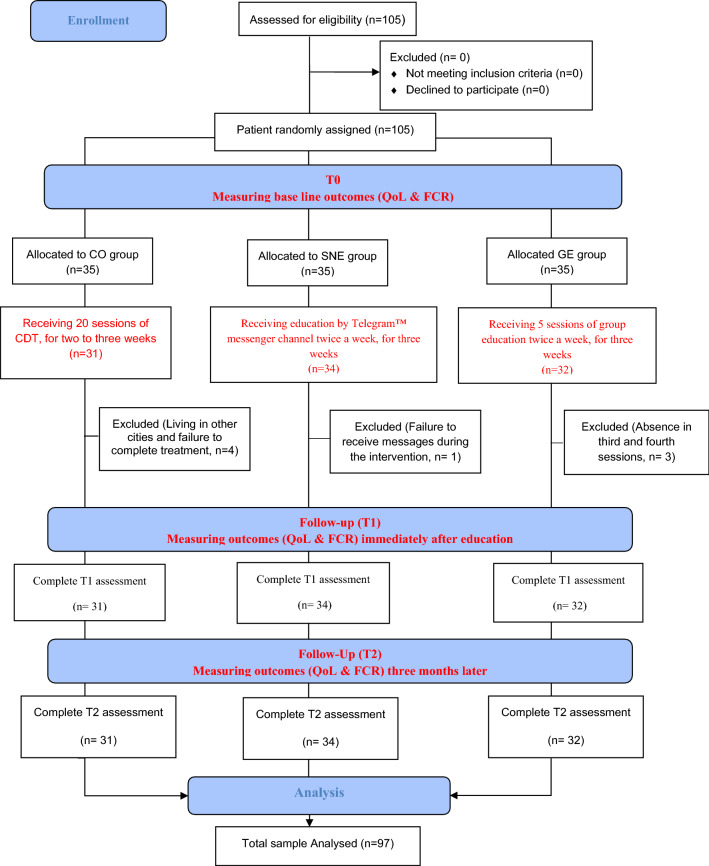
Consort diagram: recruitment and eligibility screening, randomization, follow-up, and analysis

Interested eligible patients were enrolled randomly by the blinded researcher, KH, from late September 2017 to late June 2018 at Seyed_Khandan rehabilitation center in Tehran, Iran. After obtaining written consent, patients were randomly assigned to one of the following groups: group-based education (GE), social network-based education (SNE), and control (CO). All patients underwent routine lymphedema treatments, including 20 sessions of CDT, a brochure on the care and prevention of lymphedema and a CD for rehabilitation exercises. This treatment was provided in two phases of acute and maintenance. In the acute phase, treatment was provided every day for 2–3 weeks in the clinic, and the patient at home performed the maintenance phase. In the educational groups, the outcomes of FCR and QoL were measured before the beginning of the study (T0), immediately after the completion of the three weeks of the intervention (T1), and three months later (T2). In the CO group, the outcomes of FCR and QoL were measured before the start of treatment, immediately after the end of the acute phase of treatment, and three months later. The researcher's phone number was provided to the patients to answer their questions during the study course of 3 months if any. Patients were not contacted during the three months to prevent potential bias and provision of additional information to some of them. Researchers reminded the patients of reviewing the contents and using the Telegram™ messenger by SMS every week. Blinding was not possible due to the design of the study and different treatment modalities used for each group. To prevent information contamination, the clinic staff arranged appointments for the GE group in the morning and for the other groups in the afternoon and evening to receive their treatment at the clinic. Furthermore, the participants were briefed on the study design and were asked not to share any information with others. BMI, age, and education level were measured as confounding factors (Fig. 1).

### Intervention

Based on lymphedema self-management behaviors, the educational content was designed after a review of the literature and approved by three faculty members of the Faculty of Nursing and Midwifery: JM, AK, and NE. Educational material was presented in five sessions consisting of four sessions of lymphedema self-management education and one session of stress management strategies. In the lymphedema self-management session, five skills were trained to consist of problem-solving and decision-making, using resources, applying personalized cares, cooperating with the treatment team, and sharing skills with caregivers. All of the five skills concentrated on lymphedema self-management.

#### Group education

The researcher moderated five sessions of 60 to 90 min twice a week, held in the form of face-to-face group discussions and Q&A in groups of 5 in a quiet room in the clinic. After the end of the sessions, a CD of the educational content was provided to the subjects.

#### Social network-based education

After the SNE group reached the desired sample size, a channel was created in the Telegram™ messenger called “Lymphedema Self-Management Education,” and then all SNE group subjects were invited to the channel by the researcher. Educational content was uploaded on the channel twice a week for three weeks. It was presented to the channel as 20 audio and photo messages at different times of the day. After uploading the content, subjects were asked to either notify the channel admin of receiving the content or express their opinion to ensure that everyone received the messages. In this group, the educational content was delivered exclusively through the channel created. The control group received a CD of the educational material after the study.

### Outcomes

1. Quality of life: QoL was measured with the Persian version of the Lymphedema Life Impact Scale (LLIS).
2. Fear of cancer recurrence: FCR was measured with Fear of Progression Questionnaire-Short Form (FoPQ-SF).

### Instrument

1. Demographic information form: This researcher-made form included questions about age, education level, marital status, employment status, BMI, type of surgery, duration of lymphedema, severity of lymphedema, and stage of cancer.
2. LLIS: It is an 18-item questionnaire with physical, functional, and psychosocial subscales. Items are scored based on a Likert scale from 1 (no impairment) to 5 (severe impairment) and the sum of the scores make up the total score. The total score and the scores of each subscale are a percentage ranging from 0 to 100. Lower ratings indicate less impairment, while higher scores show a more inferior status and a higher impairment in QoL. Psychometric properties of the Persian version of LLIS had been studied on a sample of Iranian breast cancer lymphedema patients by Haghighat et al. [27]. It had a relatively good construct validity and reliability. The internal consistency of the questionnaire and its subscales assessed by Cronbach’s alpha coefficient were 0.873, 0.854, and 0.884 for physical, psychosocial, and functional subscales, respectively. No threshold has been defined for QOL measured by LLIS.
3. FoPQ-SF: It is a 12-item questionnaire based on the fear of the cancer recurrence questionnaire. Items are scored based on a Likert scale from 1 (never) to 5 (always). The total score is the sum of scores of the items and 60 at maximum. The score of 46.5 is considered as the cut-off point for high FCR. The validity and reliability of the Persian version of this questionnaire were investigated by Mohajel Aghdam et al. [28]. The face and content validity of the translated questionnaire were determined by 15 academic members from Tabriz University of Medical Sciences. The internal consistency of the questionnaire was established by Cronbach's alpha coefficient (0.87) after a pilot study on 20 cancer patients.

### Sample size

Sample size was calculated for QoL changes due to intervention. Mean QoL was compared among the three groups using the $n=\frac{{\varvec{\uplambda}} }{\Delta }$ equation with 95% confidence level and 80% of test power, and the λ value of 9.64 from the table 3.4.1 from "Sample Size Calculations in Clinical Research" book [29] for the three groups. According to similar previous studies [30, 31], and assuming the QoL score difference obtained by self-care with two individual and group methods as 7.8 ± 13.6, the value of Δ harm is 0.32. After the replacement of this value in the related equation, the sample size in each group was obtained as 31 and considering 15% sample loss, the final size was estimated to be 35 for each group. The total sample size was thus 105 people.

### Randomization

Random blocks of three were used for randomization. Before the intervention, a statistician prepared 35 sets of ABC, BAC, etc., modes from numbers 1 to 35 using the MS Excel™. Every three recruited patients were assigned to one of the groups by the researcher. The letters A, B, and C were each symbol of one of the study groups.

### Statistical methods

Chi-square and one-way ANOVA tests assessed the difference in demographic and clinical variables’ frequency between three groups. Kolmogorov–Smirnov test confirmed the normal distribution of QoL and FCR variables, so parametric tests were used. Mixed-model ANOVA was applied to study the main effect of time and groups and interaction of them on outcomes after checking its assumptions in data. The variation of QoL and FCR values after three months could present the prolonged impact of intervention. Thus, the difference of outcomes at the end of the study was compared by ANCOVA test and adjusting the baseline values and group effect. The statistical significance was set as *P* < 0.05. Statistical analysis was performed by IBM SPSS Software version 20.0 and the statistical significance level of 0.05.

## Result

A few participants were excluded as follows: four subjects in the CO group (due to living in cities far from the clinic), three in the GE group (due to absenteeism in sessions 3 and 4 of education), and one in the SNE group (due to not receiving messages during the intervention). A total of 97 subjects completed the questionnaires at T1 and T2 stages. Most patients were married, educated, and with grade II—cancer and stage II of lymphedema. No significant difference was noticed among the three groups in terms of demographic characteristics (Table 1).

**Table 1:** Demographic characteristics of participants and comparison among the groups

Variable	GE N (%)	SNE N (%)	CO N (%)	*P*-value
Marital status				0.107
Single/divorced/widowed	5 (15.6)	11 (32.4)	14 (12.9)	
Married	27 (84.4)	23 (67.6)	27 (87.1)	
Educational level				
Diploma/under diploma	19 (59.4)	17 (50.0)	16 (51.6)	0.721
University	13 (40.6)	17 (50.0)	15 (48.4)	
Occupation				0.4
Employed	6 (28.6)	11 (52.4)	4 (19.0)	
Housekeeping	19 (33.3)	17 (29.8)	21 (36.8)	
Retirement	7 (36.8)	6 (31.6)	6 (31.6)	
Type of surgery				0.930
Modified radical mastectomy	18 (34.6)	18 (34.6)	16 (30.8)	
Breast preservation	14 (31.1)	16 (35.6)	15 (33.3)	
Grading of breast cancer				0.628
I	2 (33.3)	2 (33.3)	2 (33.3)	
II	15 (26.8)	22 (39.3)	19 (33.9)	
III/IV	15 (46.9)	10 (29.4)	10 (32.3)	
Severity of lymphedema				0.373
I	4 (12.5)	8 (23.5)	8 (25.8)	
II/III	28 (87.5)	26 (76.5)	23 (74.2)	

	Mean (SD)	Mean (SD)	Mean (SD)	*P*-value
Age	52.47 (10.62)	50.44 (8.81)	50.23 (8.90)	0.581
BMI (kg/m^2^)	28.04 (5.07)	28.41 (5.10)	28.35 (4.52)	0.947
Duration of lymphedema (month)	6.22 (3.86)	7.50 (3.51)	7.26 (3.34)	0.315

Comparison of QOL changes during the study has been demonstrated in Table 2. The mixed-model ANOVA results showed that the main effect of the group on total LLIS or its subscales was not significant. In contrast, the time transition had significant correlation with the mean scores of the total LLIS (*P* = 0.007), psychosocial (*P* = 0.038), and functional (*P* = 0.024) subscales. Pairwise comparison of total and functional LLIS in different time status showed that the *P*-value between T0/T1, T0/T2, and T1/T2 was 0.1, 0.015, and 0.062 and 0.1, 0.047, and 0.151, respectively. There was no significant correlation between different time intervals on the psychological subscale in T0/T1 (*P* = 0.1), T0/T2 (*P* = 0.058), and T1/T2 (*P* = 0.175) comparisons. Mixed-model ANOVA analysis of LLIS changes during three months of the study showed the interaction between group and time on functional LLIS was statistically significant (*P* = 0.017), even though multiple comparisons of study groups showed no significant correlation between GE/SNE (*P* = 0.892), GE/CO (*P* = 0.553), or SNE/CO (*P* = 0.818) groups.

**Table 2:** Comparison of QoL among the groups on T0, T1, and T2

LLIS in times of evaluation	Groups						Main effect		Interaction	ANCOVA test			
	GE		SNE		CO								
	Mean %	SD	Mean %	SD	Mean %	SD	P*	P**	P***	P****	GE/SNE	GE/CO	SNE/CO
Total													
T0	0.33	0.15	0.32	0.19	0.36	0.24	0.007^†^	0.359	0.097	0.055			
T1	0.31	0.17	0.31	0.19	0.37	0.21							
T2	0.24	0.13	0.31	0.19	0.34	0.22							
Physical													
T0	0.33	0.14	0.29	0.20	0.36	0.24	0.116	0.537	0.248	0.101			
T1	0.31	0.17	0.31	0.20	0.35	0.21							
T2	0.27	0.15	0.30	0.21	0.32	0.21							
Psychosocial													
T0	0.23	0.25	0.27	0.27	0.34	0.31	0.038^†^	0.103	0. 349	0.001^†^	0.004^†^	0.045^†^	0.392
T1	0.21	0.24	0.25	0.25	0.34	0.30							
T2	0.14	0.15	0.26	0.23	0.28	0.28							
Functional													
T0	0.40	0.25	0.41	0.24	0.40	0.27	0.024^†^	0.582	0.017^†^	0.009^†^	0.046^†^	0.001^†^	0.140
T1	0.39	0.26	0.36	0.24	0.43	0.24							
T2	0.28	0.17	0.37	0.22	0.41	0.26							

^†^Significant at the *P* < 0.05 level

P* indicates within-group effect (time effect)

P** indicates between-group effect (group effect)

P*** indicates the interaction effect (group* time effect)

P**** indicates ANCOVA test (comparing the outcome between groups after adjusting the baseline value and group effect)

The effect of intervention at the end of the study was evaluated by ANCOVA test. The LLIS score of three groups was compared at T2 time after adjusting the LLIS score at T0 and group effect. Post hoc analysis showed that significant changes in psychosocial (*P* = 0.001) and functional mean scores (*P* = 0.009) were due to further reduction of impairment in the GE group.

Table 3 represents the results of mixed-model ANOVA on FCR during the study in GE, SNE, and CO groups. The mean score of FCR changed from 34.4 to 31.8 in the GE group, from 36.3 to 35.6 in the SNE group, and from 36.6 to 35 in the CO group. Results indicated that the main effect of time and group was not significant (*P* = 0.084, *P* = 0.380, respectively). Interaction of time and group effect on FCR mean score changes did not show significant correlation, too (*P* = 0.568).

**Table 3:** Comparison of FCR among the groups on T0, T1, and T2

Fear of cancer recurrence in times of evaluation	Groups mean (SD)			P*	P*	P***	P****
	GE (*n* = 32)	SNE (*n* = 34)	CO (*n* = 31)				
T0	34.37 (12.28)	36.28 (12.43)	36.60 (11.12)	0.520	0.568	0.380	0.084
T1	31.78 (11.01)	36.23 (12.24)	36.29 (10.31)				
T2	31.81 (11.92)	35.56(12.62)	35.03(11.88)				

P* indicates within-group effect (time)

P** indicates between groups effect (groups)

P*** indicates the interaction effect (group* time)

P**** indicates ANCOVA test (comparing the outcome between groups after adjusting the baseline value and group effect)

**Fig. 2:**
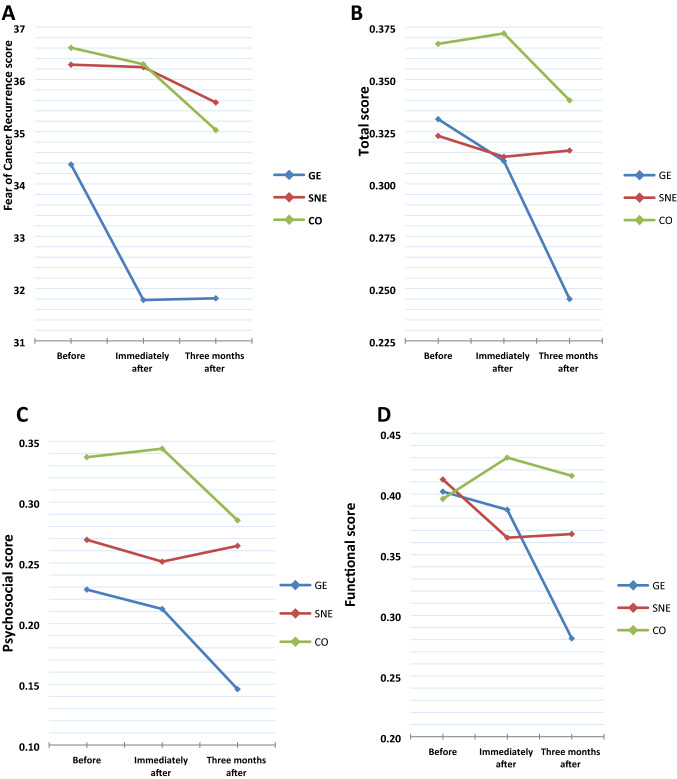
Effect of the interventions on **a** FCR, **b** total score, **c** psychosocial score, and **d** functional score of LLIS during the study

Comparison the FCR scores at the end of the study, after adjusting the baseline value and group effect by ANCOVA test, did not show a significant difference, too (*P* = 0.520). The impact of the intervention on FCR, total score, psychosocial score, and functional score of LLIS scale is demonstrated in Fig. 2.

## Discussion

This research was one of the first studies to examine group-based and social network-based education simultaneously in educating patients with lymphedema. Evidence from this study showed that lymphedema self-management group-based education was more effective than social network-based training in improving functional and psychosocial domains of quality of life after three months. In spite of the significant interaction of time and group effect on the functional subscale, no significant correlation was noticed between each two groups. There was a higher reduction of the FCR score in the GE group compared to SNE and Co arms, without any significant correlation over time among the three groups.

The study population included women with lymphedema, of whom 53.5% had a high school diploma, and 79.4% were married, their mean age was 51 years, and their mean BMI was 28. In a study by Melam on women with BCRL, most participants were educated, married, and with a mean age of 56 years [32]. The demographic characteristics of patients in this study appear to be similar to the populations studied in previous studies [33, 34].

In the present study, three months after the intervention, group-based education was more effective than social network-based training in improving the psychosocial domain. Meanwhile, Compen et al. compared the effect of routine treatment and mindfulness-based cognitive therapy with Internet-based and face-to-face methods combined with group meetings on mental tension in cancer patients. They reported both Internet-based and face-to-face approaches, combined with group sessions, were effective in the improvement of FCR, mind rumination, mental function, mental health and QoL, compared to the routine treatment [19]. This difference can be attributed to our research populations, which were women with lymphedema. Although their teaching method is similar to ours, lymphedema, with its impact on the ability to do everyday tasks, impairs writing, dressing, and returning to normal activities in women, which is associated with psychosocial disturbances [35]. It is expected that the reduction in symptoms and dysfunction will improve QoL. Three months after the intervention in our study, QoL and psychosocial status improved, while dysfunction decreased. Therefore, self-management education has been effective in improving QoL, which was more effective in the GE group. The group-based method allows women to meet peers and receive positive social support and develops their belief in self-efficacy in controlling symptoms associated with the disease through their observational learning [36–38].

Although the mean score of FCR was not high in our study population, teaching stress management methods reduced the FCR scores in both intervention groups three months later. No similar evidence was found with investigation of the fear of recurrence in lymphedema patients. So, the interfering with FCR intensity in this study population was not easy. The authors concluded that referring patients to a reputable lymphedema treatment center may have been useful in developing the patients’ confidence, reducing their health problems, and decreasing the level of fear of cancer recurrence.

In a study that investigated the effect of group-based cognitive-behavioral therapy on reducing FCR in cancer survivors, the group-based method was effective on FCR but did not affect QoL and its domains [39]. It appears that lymphedema creates a deeper FCR in women since, with each recurrence of lymphedema, FCR is also activated [10]. Considering that the present study investigated FCR in lymphedema for the first time, further studies are necessary to investigate FCR in lymphedema in collaboration with experts from other disciplines.

Besides, the similarity of FCR changes in all three groups without any significant statistically and clinically difference, maybe a confirmation of the greater efficacy of lymphedema clinical care and dilution of the intervention effect. Maybe more sessions of stress management education and cognitive-behavioral therapy are needed to provide larger effect sizes. It is suggested that this issue is addressed in future studies.

The mean score of functional and psychosocial domains and total score of LLIS decreased in the SNE group, but this decrease was not significant compared to the GE and CO group. Valle investigated the effectiveness of Facebook™-based behavioral intervention on the physical activity of cancer survivors and reported an increase in the duration of physical activity per week in the intervention and control groups three months after the intervention, which was slightly higher in the Facebook™-based group, while there was no significant difference between the two groups in terms of QoL [31]. As shown in Fig. 2, in our study, social network-based education was effective in the short term. Perhaps this was due to the law in Iran, which banned Telegram™ messenger while this study was being run, which restricted patients' access to the content uploaded during the three-month follow-up period. However, researchers tried to compensate for this restriction by sending messages through WhatsApp™ messenger. WhatsApp Messenger is a freeware messaging that allows users to send text messages and voice messages and share images, documents, and other media. Users could initially communicate only with individuals or informal groups on it [40]. The use of online social networks to change or improve behavior is still in its early stages. A lot of research is required to optimize interventions and increase the efficiency of these networks, especially in maintaining behavioral changes in the long run. Our study results on lymphedema and the use of various educational methods can provide useful evidence for further studies.

There was a significant time effect on total LLIS, psychosocial, and functional subscale changes in three groups of study. This main effect was mostly due to more considerable variations of outcome after three months of study compared to baseline values. ANCOVA showed similar results and a significant decrease in the psychosocial and functional impairments after three months of education in GE and SNE groups, with a higher reduction rate in the GE group. It can be considered as an advantage of this study that the educational effect was persistent after three months of intervention. In other studies that have examined the impact of Web-based or Internet-based interventions on QoL of cancer survivors, social performance has not changed significantly after interventions [41–43]. Although these methods and social network-based methods are not ineffective and they have a high rate of patient participation due to their ease of access, one of their biggest challenges is low treatment adherence, which affects treatment effectiveness [19]. As a limitation, patients might have learned the educational content but they did not know its application accurately. This defect of knowledge may affect the measured outcomes. This limitation might affect the results of the interaction of time and group on functional quality of life in mixed-model ANOVA, too. In spite of the significant interaction effect (*P* = 0.017) in this correlation, no significant association was noticed in multiple comparisons of groups. This may be due to the limitation of transferring knowledge in a short interval time. Future researches by considering probable confounders such as virtual health literacy, different educational packages, and so on may provide beneficial pieces of evidence.

In spite of the lower loss to follow-up in the SNE group compared to other groups, reminding the patients of reviewing the contents weekly and providing a functional and useful educational package, the reduction of scores in this group was not significant. Therefore, considering the benefits of social networks, more studies are required on the adherence rates of participants. Due to the particular design of the present study, the adherence rate could not be appropriately investigated. Its evaluation may be suggested for future researches, considering that in real-life situations vs research and lab-based environments, adherence cannot always be measured.

QoL significantly improved using lymphedema self-management education through group-based education compared to social network-based education and routine treatments. Therefore, in-person training with the participation of peers in this population of cancer survivors still has a special place. Of course, given the widespread use of social networks such as Telegram™ and the potential of these networks to change patient’s behavior, their position can be enhanced in education. According to the Iran National Cyberspace Institute's statistics, each Iranian is a member of 13 public channels in Telegram™ and receives 100 posts per day on average [44]. The promotion of social network status as an environment for education requires promoting people’s awareness, increasing the attractiveness of educational contexts and more research into other diseases. It seems that shortening the online or in-person communication interval time with patients may be a good strategy for increasing the efficacy of social networks' role in education. Other treatment staff, such as midwives, breast care nurses, and physiotherapists who have more communication with women, can act as online or in-person supporters for the treatment of women with lymphedema. The increasing number of lymphedema patients, the lack of needed lymph therapy centers in Iran, the costs of in-person education such as personnel costs, traveling costs of patients and health providers, and costs of consultation place are essential issues in cost-effectiveness evaluation of those two educational interventions. Considering higher prices of GE compared to SNE in most of those issues versus near to equal effect sizes of two responses may introduce the virtual education as n prior intervention compared to in-person training. Designing a precise cost-effectiveness study for assessment of this assumption would be beneficial. Although this study was conducted in one clinic, given it is a referral clinic and accepts patients referred from most parts of the country, the results of this study are mostly applicable to women with lymphedema in Iran.

## Conclusion

Both methods of group-based and social network-based education of lymphedema self-management improved QoL and reduced the disruption due to lymphedema in life. However, the group-based method had more significant effects on the psychosocial and functional domains. Group-based and social network-based education methods reduced FCR, which was not significant. According to the results of this study, physicians can preserve the effects of lymphedema treatment by educating patients and using various educational methods such as virtual education or periodic workshops and increase patients' motivation to adhere to self-management behaviors.

## Data Availability

The datasets generated and analyzed during the current study are available through contact with the corresponding authors: sha_haghighat@yahoo.com.

## Abbreviations

BCRL: Breast cancer-related lymphedema
QoL: Quality of life
FCR: Fear of cancer recurrence
GE: Group-based education
SNE: Social network-based education
CO: Control group
CDT: Complete decongestive therapy
